# Malignant Lymphoproliferative Disorders of the Oral and Maxillofacial Region: Report of Two Institutions

**DOI:** 10.3389/froh.2022.802555

**Published:** 2022-02-03

**Authors:** Andres Flores-Hidalgo, Alec Bankhead, Valerie Murrah, Ricardo Padilla

**Affiliations:** ^1^Department of Surgical Sciences, Division of Oral and Maxillofacial Pathology, East Carolina University School of Dental Medicine, Greenville, NC, United States; ^2^Department of Surgery, University of Cincinnati College of Medicine, Cincinnati, OH, United States; ^3^Division of Diagnostic Sciences, University of North Carolina at Chapel Hill Adams School of Dentistry, Chapel Hill, NC, United States

**Keywords:** lymphoma, North Carolina, plasma cell myeloma, World Health Organization, oral cavity

## Abstract

**Background:**

Report of the incidence of malignant hematologic neoplasms in the oral cavity according to the experience of the two oral and maxillofacial pathology institutions in North Carolina, USA.

**Methods:**

A 10-year retrospective review was carried out in the records of patients at ECU SoDM and UNC Adams SoD. Age, sex, location of the lesion, clinical impression, initial diagnosis, and the expression immunohistochemical and molecular markers were recorded for each subject. All diagnoses were reviewed according to the 2016 revision of the WHO classification of lymphoid neoplasms.

**Results:**

A total of 318 records from both institutions were reviewed. Seventy males and 68 females with an average age of 60.7 comprised the study population. The most common neoplasm encountered was plasma cell myeloma, followed by diffuse large B-cell lymphoma, B-lymphoblastic lymphoma, and follicular lymphoma. We encountered primarily intraosseous tumors, with the posterior mandible and posterior maxilla being the most common locations. Twelve cases were identified initially as a periapical radiolucency.

**Conclusion:**

Our findings are concurrent with the existing literature regarding epidemiologic data. However, the type and location of tumors encountered do not, as the most common lymphoma in the oral cavity is diffuse large B-cell lymphoma, typically present in soft tissue. To aid in diagnosis and treatment, the collection of data should continue over time so that eventually, a more specific diagnostic profile of North Carolina residents with these neoplasms can be made.

## Introduction

There has been a recent yearly increase in the incidence of Non-Hodgkin Lymphomas (NHL), which currently represents 4% of all cancers in the United States [1]. While there is abundant existing literature reporting the incidence of lymphoproliferative disorders (LPDs) in the head and neck, few publications have data specifically for the oral and maxillofacial region. For dental providers this information is critical, as they must be able to identify, diagnose, and treat these malignancies based on current criteria in order to improve patient outcomes.

Proper diagnosis and classification is an essential step in the successful treatment of oral and maxillofacial malignancies. With improvement in diagnostic techniques and an increasing body of evidence, classification systems are periodically updated. The World Health Organization (WHO) revised its 2008 classification of lymphoid neoplasms in 2016 to provide a more accurate diagnosis, classification, and management of these malignancies [2].

This study aimed to collaboratively report the incidence of malignant lymphoid neoplasms of the oral and maxillofacial region according to the experience of the two oral and maxillofacial pathology biopsy services in dental schools in the State of North Carolina. With this data, we hope to increase awareness of the incidence of oral cavity lymphoid malignancies in our State and improve future treatment outcomes based on these diagnostic changes.

## Materials and Methods

This report is a retrospective cross-sectional study of biopsy specimen records diagnosed as any of the malignant lymphoid neoplasms. The study protocol was registered, reviewed, and approved by the East Carolina University and the University of North Carolina at Chapel Hill Institutional Review Boards under the numbers 19-000304 and 18-3104, respectively. All study procedures were performed in accordance with the Declaration of Helsinki and Research Committee Regulations.

A diagnosis-based search in the electronic health records of both institutions was performed to identify all cases of lymphoproliferative disorders of the oral and maxillofacial region. The study population consisted of all patients diagnosed with oral cavity malignant LPDs at East Carolina School of Dental Medicine and the UNC Adams School of Dentistry from July 1, 2005, to December 31, 2018. Our inclusion criteria selected cases that were diagnosed as malignant lymphoid neoplasm in the oral and maxillofacial region, and in which the diagnosis was confirmed with a formal consultation by a Board-Certified Hematopathologist at the Department of Pathology. We excluded from the study benign or reactive diagnoses (such as EBV-related benign or reactive proliferations), cases with no official report from hematopathology, or those outside of the specified study period.

Age, sex, location, clinical impression, pertinent medical history, diagnosis, immunohistochemical, and molecular panels were recorded for each case.

## Results

Our search of both institution's databases yielded 138 cases that met our inclusion criteria. The age of the population ranged from 6 to 93 years, with a mean age of 60.7 (median 58.5). The gender distribution was 70 males and 68 females. Significant available medical history at the time of diagnosis included HIV, EBV, vasculitis, multiple myeloma, Crohn's disease, myasthenia gravis, rheumatoid arthritis, previous breast cancer, previous non-Hodgkin lymphoma, previous plasma cell myeloma, previous chronic lymphocytic lymphoma (CLL), and previous lung cancer.

The most common diagnosis in our cases was plasma cell myeloma/multiple myeloma (MM), comprising 28.9%, with the majority being kappa-restricted neoplasms. Multiple myeloma was followed in incidence by Diffuse Large B-Cell Lymphoma (DLBCL), Plasmablastic Lymphoma (PL), Follicular Lymphoma (FL), and other lymphoid neoplasms. A comprehensive list of all diagnoses, incidences and percentages of our cases is presented in Table 1. The most common location at initial diagnosis was the posterior mandible, followed by the buccal mucosa, palate, posterior maxilla, gingiva, and other locations listed in Table 2. We also observed a slight increase of 10% in the incidence of LPDs submitted to our biopsy services over time over time (2005 to 2018); being the year 2017 the year with more reported cases. The increased incidence was mainly observed in cases of older Caucasian (non-Hispanic whites).

**Table 1:** Incidence of malignant lymphoid neoplasms in the oral cavity by diagnosis rendered.

**Diagnoses**	**Incidence (%)**
Plasma cell myeloma (40)	28.9
Diffuse large B-Cell lymphoma (15)	10.8
Plasmablastic lymphoma (13)	9.4
Follicular lymphoma (12)	8.6
MALT lymphoma (10)	7.2
EBV+ Lymphoproliferative process, NOS (6)	4.3
Mantle cell lymphoma (5)	3.6
B-Lymphoblastic lymphoma (5)	3.6
Langerhans cell histiocytosis (4)	2.8
Burkitt lymoma (4)	2.8
Lymphoplasmacytic lymphoma (2)	2.1
Acute myeloid leukemia (1)	0.7
B-cell lymphoma, not otherwise specified (21) • High-grade B-cell lymphoma (3) • Mature B-cell lymphoma (14) • Low-grade B-cell lymphoma (2) • B-cell lymphoma [NOS] (2)	15.0

**Table 2:** Intraoral incidence of malignant lymphoid neoplasms according to site.

**Locations**	**Incidence (%)**
Posterior mandible (37)	26.8
Buccal mucosa (21)	15.2
Palate (20)	14.5
Posterior maxilla (10)	7.2
Gingiva (10)	7.2
Lip (10)	7.2
Anterior maxilla (9)	6.5
Parotid (7)	5.1
Anterior mandible (4)	2.9
Floor of mouth (4)	2.9
Tongue (3)	2.2
Maxilla and mandible combined (1)	0.7
Chin (1)	0.7
Parotid lymph node (1)	0.7

**Figure 1:**
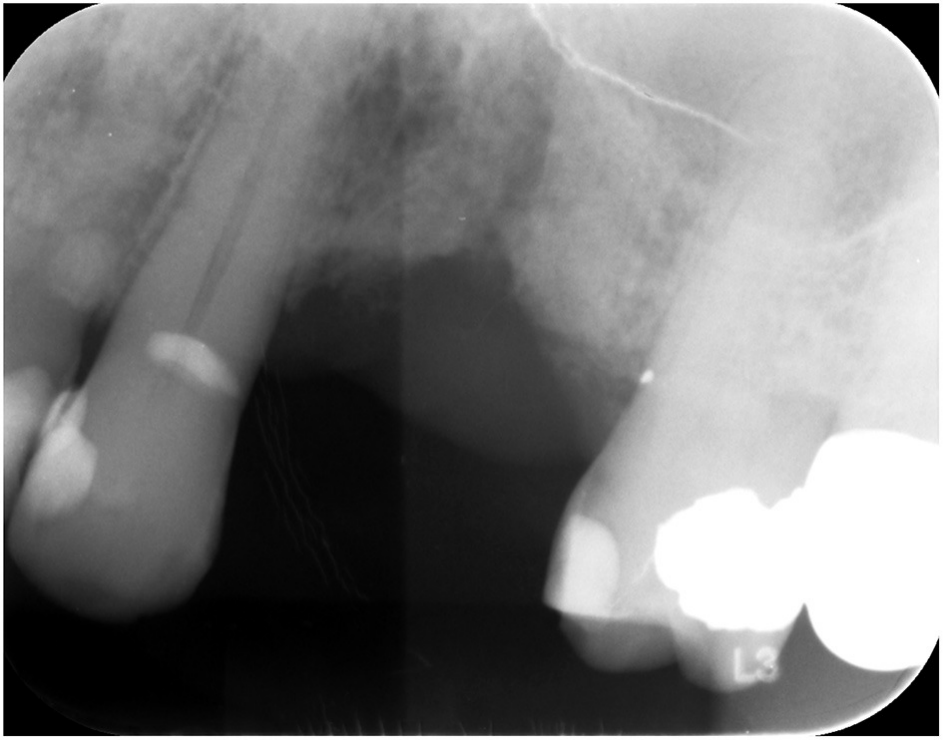
Ill-defined radiolucent lesion, persistent after dental extraction.

Twelve cases were initially identified after apicoectomy of persistent periapical lesions from failed endodontic treatment with persistent periapical radiolucency or after dental extraction (Figure 1). These lesions were primarily encountered in the posterior mandible and maxilla. Said specimens were usually submitted with a clinical impression of a periapical cyst, persistent periapical radiolucency, periapical granuloma, or residual cyst in those cases with a persistent radiolucency after dental extraction. The more common diagnoses encountered with such clinical and radiographic presentation were mostly MM, followed by DLBCL, and low-grade B-cell lymphomas. Such cases were also encountered in older Caucasian and African American males in the 4–5th decade of life.

Of the 138 cases we identified, 15 were reclassified according to the 2016 WHO classification of lymphoid neoplasms. This process entailed only nomenclature changes, per new guidelines, but did not yield any diagnostic or prognostic significance for the involved patients. Among these cases, the most common diagnoses were DLBCL, low-grade b-cell lymphomas, and MM.

## Discussion

The first aim of this study was to document the incidence of malignant hematolymphoid neoplasms in the oral and maxillofacial region based on the collaborative experience of the two dental schools in North Carolina. Based on the updated 2016 WHO lymphoproliferative diseases classification system, we hypothesized that some diagnoses from our cases would need to be reclassified. Our second aim was to acknowledge this change and evaluate if some cases' reclassification would alter treatment outcomes and prognosis.

**Figure 2:**
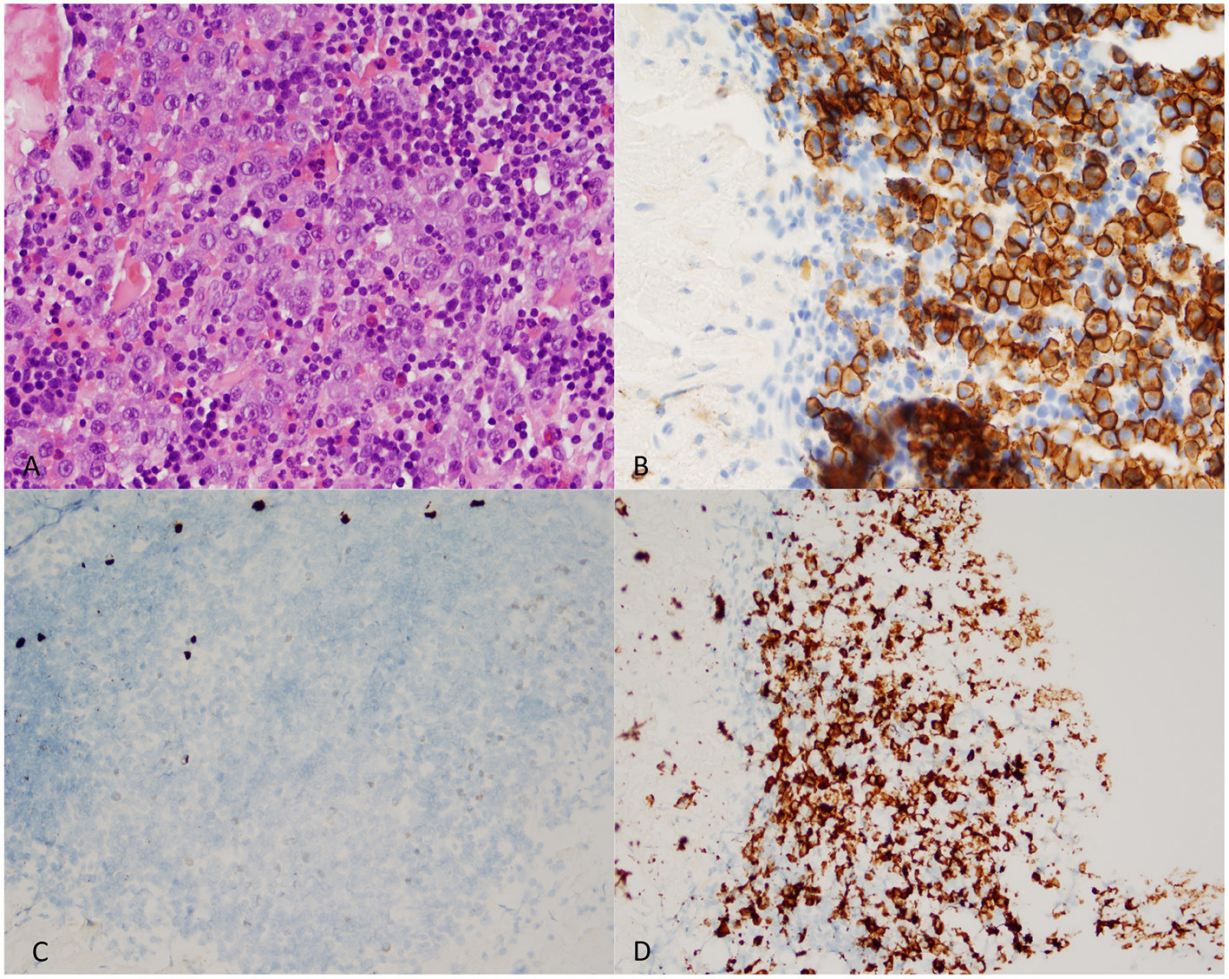
Representative microscopic sections of a lambda-restrictive Plasma Cell Neoplasm (multiple myeloma) encountered in the posterior mandible of a 68-year-old male. **(A)** Aberrant plasma cells within a background of lymphocytes (Hematoxyline and eosine - 400X magnification). **(B)** CD138 Immunoexpression in cell population of interest (400X magnification). **(C)** Low-power field of *in-situ* hybridization shows a sparse cell population with kappa-light chain expression (Kappa ISH - 200X magnification). **(D)** Increased presence of Lambda-restricted cell population (Lambda- ISH - 200X magnification).

A total of 318 cases of lymphoproliferative neoplasms were reviewed, yielding 138 that met our inclusion criteria. The most common diagnosis in our cases was MM (Figure 2). The three most common locations for all neoplasms were the posterior mandible, buccal mucosa, and palate. Fifteen of our original diagnoses were reclassified according to the new classification system, but no significant difference in prognostic value or treatment was estimated when compared to the original diagnoses. A small number of cases (15%) were generically diagnosed as “B-cell lymphoma” after a formal consultation with hematopathology. This broad, non-specific diagnosis was either based on clinical correlation limitations or out of necessity due to the small tissue sample size. If the tissue sample submitted was too small to perform a full panel of ancillary studies necessary for further typification, the diagnosis of B-cell lymphoma was provided, and rebiopsy or biopsy of a different site was advised. In addition to IRB limitations, the scope of our project precluded us from further searching the patient's medical records for a more definitive diagnosis in these cases.

Literature providing information on the incidence of lymphomas of the head and neck has been presented. Walter et al. [3] retrospectively analyzed records from an Oral and Maxillofacial Surgery Department in a German Medical Center over 40 years and found that more than 50% of the head and neck lymphomas were initially identified in the oral cavity. In a similar design, a retrospective 10-year study by Niemec et al. [4] analyzed location, histological type, and incidence of lymphomas in the head and neck due to the increasing yearly incidence of NHL worldwide. To the best of our knowledge, a retrospective reclassification of lymphoid neoplasms of the head and neck as presented in our study has not been performed similarly in the English literature.

There were similar age and sex characteristics of our patient population (average age 60.7 with a male-to-female ratio close to 1:1) compared to existing institutionally reported retrospective studies [3, 4]. However, one systematic review presenting oral cavity lymphomas showed a male predilection and a less specific age frequency of head and neck LPDs in those aged 50 years or older [5]. This same distribution of age and sex is consistent with findings presented by Triantafillidou et al. [6] with a similar number of cases. Most reports in the literature, including this study, present a wide range of ages at the time of diagnosis, with most oral cavity malignancies diagnosed in the elderly.

In our data, diagnoses of oral and maxillofacial LPDs are of majority B-cell origin (96.3%), following their incidence reported in the literature [3, 4, 6, 7]. The most commonly reported oral cavity hematolymphoid malignancy is DLBCL, followed by follicular lymphoma, mantle cell lymphoma, Burkitt lymphoma, marginal zone lymphoma, B-lymphoblastic lymphoma, and other less common lymphoproliferative neoplasms [4–6]. DLBCL has the highest incidence of all oral cavity lymphomas at 25–50% [5, 6, 8]. Diffuse large B-cell lymphoma was second in incidence in our study, comprising 10.8% of the diagnoses. The only hematolymphoid neoplasm with a higher incidence in our series was plasma cell myeloma, accounting for 28.9% of our cases. This difference may be due to the fact that our study also included cases of the jaws, while some series in the literature may exclude intrabony lesions and report only those that are mucosal-based. If we excluded intrabony lesions, our data also support DLBCL as the most frequent hematolymphoid neoplasm of the oral mucosa.

The most commonly reported locations of oral cavity lymphomas are in soft tissue, presenting most often in the salivary glands, buccal mucosa, and gingiva [5, 6, 9]. The majority (59%) of our study lesions were in osseous structures, most frequently the posterior mandible, anterior and posterior maxilla, and hard palate. Because MM was the most common neoplasm in our study, these results are to be expected considering MM rarely presents in extraosseous locations.

A limitation of this study is its retrospective design. Our reporting of the demographics and location of the lesions depended on the documentation and diagnostic accuracy of the clinical practitioners who sent their cases to our institutions, hence access to full medical and dental records was limited or impossible. For these outside cases, the majority of the collected data was found in biopsy requisition forms. In order to report the most clinically significant findings from the patient population, we only reported malignant neoplasms that presented initially in the oral and maxillofacial region within a specified period.

## Conclusion

In our 15-year retrospective study of the biopsy services of the two dental schools in North Carolina, MM was the most common malignant LPD presenting in the oral and maxillofacial region. Most hematolymphoid malignancies presented in our study were intra-bony, such as periapical radiolucencies, and submitted as failed endodontic therapy or “residual cyst” after dental extraction. This highlights the importance of submitting tissue samples for microscopic examination even if they present as persistent periapical lesions after failed root canal therapy, as prompt diagnosis and treatment of hematolymphoid neoplasms imply better patient outcomes. The collection of data should continue over time so that eventually, a more precise classification of hematolymphoid neoplasms of the oral and maxillofacial region in the population of North Carolina can be reached.

## Data Availability Statement

The raw data supporting the conclusions of this article will be made available by the authors, without undue reservation.

## Ethics Statement

The studies involving human participants were reviewed and approved by ECU University and Medical Center Institutional Review Board – 19-000304 UNC at Chapel Hill Institutional Review Board – 18-3104. Written informed consent for participation was not required for this study in accordance with the national legislation and the institutional requirements.

## Author Contributions

AF-H: data collection, analysis, and manuscript preparation. AB: data analysis and manuscript preparation. All authors contributed to the article and approved the submitted version.

## Conflict of Interest

The authors declare that the research was conducted in the absence of any commercial or financial relationships that could be construed as a potential conflict of interest.

## Publisher's Note

All claims expressed in this article are solely those of the authors and do not necessarily represent those of their affiliated organizations, or those of the publisher, the editors and the reviewers. Any product that may be evaluated in this article, or claim that may be made by its manufacturer, is not guaranteed or endorsed by the publisher.
